# Sini Decoction Ameliorates Colorectal Cancer and Modulates the Composition of Gut Microbiota in Mice

**DOI:** 10.3389/fphar.2021.609992

**Published:** 2021-03-11

**Authors:** Yishan Wang, Xiaodi Zhang, Jiawei Li, Ying Zhang, Yingjie Guo, Qing Chang, Li Chen, Yiwei Wang, Siyao Wang, Yu Song, Yongkun Zhao, Zhihong Wang

**Affiliations:** ^1^ Key Laboratory of Utilization and Conservation for Tropical Marine Bioresources, Ministry of Education, Key Laboratory for Protection and Utilization of Tropical Marine Fishery Resources, College of Fishery and Life Science, Hainan Tropical Ocean University, Sanya, China; ^2^ College of Animal Science, Jilin University, Changchun, China; ^3^ Department of Virology, Institute of Military Veterinary Medicine, Changchun, China; ^4^ Changchun University of Chinese Medicine, Changchun, China

**Keywords:** colorectal cancer, sini decoction, gut microbiota, traditional chinese medicine, mouse model

## Abstract

Sini Decoction (SND), as a classic prescription of Traditional Chinese Medicine (TCM), has been proved to be clinically useful in cardiomyopathy and inflammatory bowel diseases. However, the role and mechanism of SND in colitis-associated cancer remains unclear. This study aims to evaluate the effect of SND on colorectal cancer(CRC) symptoms and further explore the changes of gut microbes mediated by SND extract in azoxymethane (AOM)/dextran sulfate sodium (DSS)-induced CRC mice through 16 S rRNA sequencing. Our results indicated that treatment with SND extract could ameliorate the tumors' malignant degree by decreasing tumor number and size. Also, the expression levels of Cyclooxygenase 2 and Mucin-2, which are typical CRC biomarkers, were reduced compared to the CRC group. In the meantime, SND extract can upregulate CD8^+^ T lymphocytes' expression and Occludin in the colonic mucosal layer. Besides, SND inhibited the expression of CD4^+^ T cells and inflammatory cytokines in CRC tissue. According to bioinformatics analysis, SND extract was also suggested could modulate the gut microbial community. After the SND treatment, compared with the CRC mice model, the number of pathogenic bacteria showed a significant reduction, including *Bacteroides fragilis* and *Sulphate-reducing bacteria*; and SND increased the relative contents of the beneficial bacteria, including *Lactobacillus, Bacillus coagulans*, *Akkermansia muciniphila,* and *Bifidobacterium*. In summary, SND can effectively intervene in colorectal cancer development by regulating intestinal immunity, protecting the colonic mucosal barrier, and SND can change the intestinal microbiota composition in mice.

## Introduction

Colorectal cancer(CRC) is one of the most common digestive tract cancers with a high fatality rate (Favoriti et al., 2016). Studies have shown that the CRC incidence increases with the duration of inflammatory bowel disease(IBD) (Mattar et al., 2011). During IBD development, clonal evolution has begun long before the development of overt neoplasia and is probably accelerated by the repeated cycles of epithelial wounding and repair (Choi et al., 2017). Patients with IBD are considered at high risk of CRC and therefore receive anti-inflammatory treatment (Adami et al., 2016). It is essential to prevent colon cancer in the inflammatory stage and inhibit inflammation when concurrent cancer is detected. In the meantime, given the high rate of CRC recurrence accompanied by side effects in surgery and chemotherapy, there is an increasing interest in the use of complementary and alternative medicine (CAM) in which Traditional Chinese Medicine (TCM) has a vital place (Rahman et al., 2017). TCM is an ancient health care system that has been constructed for thousands of years in China, and TCM treatment modalities have been rediscovered as a popular treatment alternative to make patients feel comfortable by promoting homeostasis in recent decades. Furthermore, the increasing evidence that TCM could help relieve the CRC (Yu et al., 2015; Li, 2018) suggests that TCM has excellent colon cancer treatment potential.

Composed of Fuzi(the root of *Aconitum carmichaelii Debx*), Zhigancao(the root of *Glycyrrhiza uralensis Fisch*), and Ganjiang(the root of *Zingiber officinale Roscoe*), Sini decoction(SND) is a classic TCM prescription in "Shang Han Lun." Current chemomics and pharmacology proved that the total alkaloids, total gingerols, total flavones, and total saponins are the primary active ingredients of Fuzi, Zhigancao, and Ganjiang in SND, respectively (Yue et al., 2009; Zhang and Ye, 2009; Chen et al., 2013). However, SND exhibits substantial toxicity due to aconitine alkaloids. Therefore, it is necessary to add two other herbs to reduce toxicity (Tan et al., 2014). Evidence shows that SND can relieve UC by increasing the level of IL-10 and downregulating IL-6, IL-17, and TNF-α in mice (Zhang et al., 2020). Furthermore, SND's active components were validated to directly bind to TNF-α and exert anti-myocardial cell apoptosis effects (Chen et al., 2016). Thus, we speculated that SND is likely to alleviate colorectal cancer through its anti-inflammatory effect.

The microbiota is an integral part of the body composition, mainly distributed in the skin, oral cavity, and gastrointestinal tract. It can maintain normal physiological intestine functions and regulating immunity (Hooper et al., 2012). The colon is the site with the highest density of microbiota distribution, and microbiota imbalance can induce colonic disease (Ma et al., 2008). The intestine's microbiota is equivalent to a virtual organ and is closely related to the human body (O’Hara and Shanahan, 2006). Recently, increasing evidence has presented that gut microbiota plays an essential role in the occurrence and development of IBD and CRC (Chen et al., 2017). Yu Jun et al. found that gut microbiota and the CRC could influence each other, that microbial dysbiosis could induce CRC and accelerate its development (Yang and Yu, 2018). Fecal sample testing results from patients with CRC and healthy individuals have shown that *Enterococcus* and *Streptococcus* are increasingly present in samples from CRC patients, while the butyrate-producing bacteria *Roseburia* and *Clostridium* predominate in the control samples (Wu et al., 2009). Meanwhile, TCM can be effective by regulating the gut microbiota structure (Yue et al., 2019). Metformin and the Chinese herbal formula “Ge Gen Qin Lian Tang” may ameliorate type 2 diabetes with hyperlipidemia via enriching beneficial bacteria, such as *Blautia* and *Faecalibacterium spp* (Tong et al., 2018). Also, SND has been proved to have an excellent therapeutic effect on UC (Jian, 2010), and it can alleviate lung injury by regulating the pulmonary flora (Wang et al., 2020).

In this research, we used azoxymethane (AOM)/dextran sulfate sodium (DSS) to induce continuous inflammation and develop colorectal cancer in mice intestinal tracts to investigate further whether SND can alleviate the symptoms of cancer. Also, we evaluated the regulatory effects of SND treatment on inflammatory factors and gut microbiota structure in the development of colorectal cancer. Our primary interest is how SND regulates the gut microbiota and whether this regulation could alleviate CRC. We hypothesis SND can be a new kind of CAM to CRC.

## Materials and Methods

### Preparation of SND

The Changchun University of Chinese Medicine gifted Fuzi(*Aconitum carmichaelii Debx*), Zhigancao(*Glycyrrhiza uralensis Fisch*), and Ganjiang(*Zingiber officinale Roscoe*). Fuzi, Zhigancao, and Ganjiang were purchased in Sichuan Province, China, Ningxia Province, China, and Hebei Province, China. Boil 103 g of Fuzi, 385 g of Zhigancao, 512 g of Ganjiang (Table 1) in 2 L water for 60 min, and then boil it again 40 min after cooling. Then discard the filter residue and freeze-dry the filtrate into a dry powder (300 g).

**TABLE 1:** Ingredient composition (g/kg) of Sini Decoction.

Ingredients	Sini decoction (g/kg
Fuzi	103
Ganjiang	512
Zhigancao	385
Total	1000

### HPLC Analysis

SND extraction and HPLC analysis were conducted. Prminence-I LC-2030 Plus HPLC system (Shimadzu Corporation, Japan) was used for qualitative analysis of SND extraction. Extracts were separated by a Nanologica SVEA C18 column (250×4.6 mm5 μm). The injection volume was 5 μm. The mobile phase consisted of linear gradients of 0.1% (v/v) formic acid (A) and acetonitrile (B): 0–10 min, 25% A (v/v), 75% B (v/v); 10–20 min, 35% A, 65% B; 20–30 min, 50% A, 50% B; 30–45 min, 85% A, 15% B; 45–50 min, 25% A, 75% B; 50–60 min, 25% A, 20% B. The mobile phase flow rate was 0.5 ml/min. The column was run at 30°C. Preparation of SND sample for HPLC: ①weigh 1.000 g of Sini Decoction sample, put it into a conical flask with stopper; ②add 50 ml of methanol precisely, conduct ultrasonic treatment for 30 min, cool down, weigh; ③ make up methanol to reduce weight loss, and filter with microporous filter membrane(0.22 μm) to obtain Sini Decoction test solution. Preparation of the reference solution: take an appropriate amount of the solid of each reference substance, accurately weigh it, add methanol to prepare the mixed reference solution (Quercetin 10 ng/ml, Ammonium Glycyrrhizinate 10 ng/ml, 6-Gingerol 1.06 mg/ml, Carbenoxolone 10 ng/ml), filter through 0.22 μm microporous membrane, and refrigerate for standby. Take 10 batches of Sini decoction solution (S1 ∼ S10) and record the chromatograms. Import the chromatograms of 10 batches of Sini decoction solution into the similarity evaluation system of chromatographic fingerprints of traditional Chinese medicine (2004a Edition) for analysis. Calibrate the chromatographic peaks with a larger peak area and better resolution, with four common peaks. Establish the control fingerprint (R) with the median method, and calculate the similarity between ten batches of extracts and the control spectrum shown in Supplementary Table S1, which shows the similarity between different Sini batches decoction is good and the quality is stable.

### Animals and Experimental Protocol

Male C57BL/6 mice (6 weeks of age, 18–20 g) were purchased from Vital River Company (Beijing, China) and bred in a specific-pathogen-free (SPF) facility of the Experimental Animal Center at Jilin University, China. Experimental animal breeding and practical operations were carried out following the Public Health Service Policy on Humane Care and Use of Laboratory Animals. Animal welfare was inspected and approved by the Institutional Animal Care and Use Committee (IACUC) of Jilin University.

**FIGURE 1:**
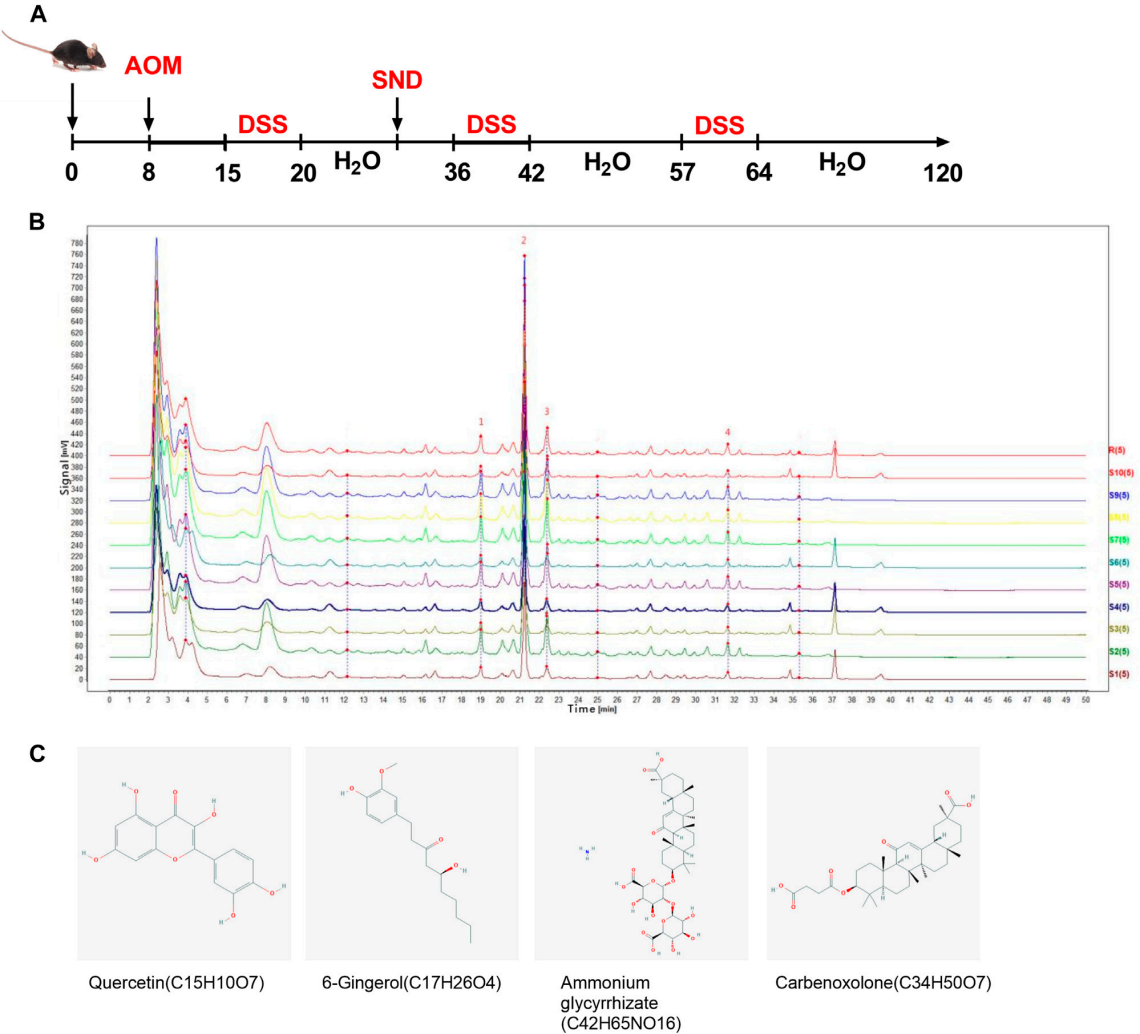
Experimental protocol and phytochemical analysis. **(A)** Animal experiment design. **(B)** HPLC chromatogram of SND recorded at 254 nm. Peaks of four primary ingredients, including (1)Quercetin, (2)Ammonium glycyrrhizate, (3)6-Gingerol, and (4)Carbenoxolone, are shown. **(C)** Structures of the four active ingredients.

The schematic of the experimental design is shown in Figure 1. Mice were allowed to acclimate for 1 week, after which they were randomly divided into four groups, with five in each group: control group (CTL), CRC model group (CRC), CTL + SND group, and CRC + SND group. 10 mg/kg AOM (Sigma) was administered to mice in the CRC and CRC + SND group by intraperitoneal injection. After regular feeding for 1 week, mice were given 2.5% DSS (TdB) in distilled water for 7 days and then were changed back to regular drinking water for 14 days. These 19 days were treated as a DSS cycle, and this process was repeated twice more. During this period, mice in the CTL and CTL + SND group were given regular water. After 3 DSS cycles, all the mice were fed generally for 2 months before sacrificing (day 120). The SND administration to the CRC + SND and CTL + SND was from day 30 to day 120 orally every day. Each mouse was given SND 0.2 ml orally. Finally, each mouse's dosage was established as 70 mg/only. The mice's body weight was recorded every day, and the colon tissue samples were collected after sacrifice. The fecal samples were collected in the last 10 days (day 110–120).

### Disease Activity Index (DAI) Assessment

The DAI was calculated by assigning well-established and validated scores for somewhat analogous parameters to human IBD's clinical presentation. The following parameters were used for calculation: 1) weight loss (0 point = none, 1 point = 1%–5% weight loss, 2 points = 5%–10% weight loss, 3 points = 10%–15% weight loss and 4 points = more than 15% weight loss); 2) stool consistency/diarrhea (0 points = normal, 2 points = loose stools, 4 points = watery diarrhea); 3) bleeding (0 points = no bleeding, 2 points = slight bleeding, 4 points = gross bleeding). The DAI has been calculated as the total of these scores: the sum of weight loss, diarrhea, and bleeding, resulting in the total DAI score ranging from 0 (unaffected) to 12 (severe colitis).

### Measurement of Colonic Phenotype

At the end of the animal experiment, mice were sacrificed by cervical dislocation under anesthesia using isoflurane. After being isolated from the abdomen, the colon tissue was cut longitudinally and flushed with the 4% cold PBS wash buffer to clean up feces and bacteria. We dry the colon with sterile gauze and measure the length and weight of the colon. Then the colonic tumors are counted, weighed, and volume measured. The proximal colon was stored in germ-free 1.5 ml centrifuge tubes, frozen in −80 C refrigerator; the distal colon was fixed in 4% paraformaldehyde overnight and embedded in paraffin routine H&E histopathologic examination. The whole tissue collection process is carried out at low temperature (0–4°C), and colon tissues' characterization was photographed.

### Histological Staining and Immunohistochemical Assessment

Routine H&E histopathologic examination was used to detect colonic tissue morphology in different groups of mice. Meanwhile, immunohistochemistry was adopted to detect inflammation-related markers and proteins according to the Histostain TM-Plus Kits (IgG/Bio, S-A/HRP, DAB, Beijing Zhongshan Goldenbridge Company, China). The colon tissue samples of mice preserved in 4% paraformaldehyde were dehydrated and embedded in paraffin. The paraffin sections were removed and then immersed in the distilled water following routine methods. Afterwards, Rinsing the paraffin sections (3 × 5 min) in PBS-T (0.01M PBS, pH 7.4: 0.02% KH2PO4, 0.29% N2HPO4, 0.02% KCl, 0.8% NaCl, 0.05% BSA, 0.05% Tween-20, 0.0015% TritonX-100) and then blocked with 3% peroxide-methanol at room temperature for endogenous peroxidase ablation. Whereafter, all the following steps of blocking, rinsing, coloration, dehydration, clearing, and mounting were carried out in a moist chamber. Anti-CD4 antibody (Abcam, ab183685), Anti-CD8 alpha antibody (Abcam, ab217344), and Anti-Occludin Polyclonal Antibody (Abcam, ab222691) were used to evaluate the immunohistochemical expression of CD4^+^ T cells, CD8^+^ T cells, and Occludin. The signals were detected by DAB (Dako, Agilent Technologies, Santa Clara, CA).

### Enzyme-Linked Immunosorbent Assay (ELISA)

Mouse colonic tissue proteins were extracted from RIPA lysis buffer (Solarbio Life Science) containing PMSF. After the protein contents were quantified by the guidance of the BCA Protein Assay Kit (Beyotime, China), the expression levels of cytokines including IL-6 (Biolegand Mouse IL-6 ELISA MAX™ Deluxe Set, B256933), IL-17 (Biolegand Mouse IL-17A/F ELISA MAX™ Deluxe Set, B255885), TNF-α (Biolegand Mouse TNF-α ELISA MAX™ Deluxe Set, B265616), and IFN-γ (Biolegand Mouse IFN-γ ELISA MAX™ Deluxe Set, B263682) were detected. All the procedures of ELISA assays followed the instructions.

### Fecal 16S rRNA Gene Sequencing-Based Phylogenetic Analysis

An Illumina MiSeq DNA sequencer performed the 16S rRNA gene sequencing at the Novogene Technology Co., Ltd. Raw sequence data were processed with the Qiime software. OTUs were picked at 97% sequence identity using cdhit in Qiime wrappers, and a representative sequence was then chosen for each OTU by selecting the most abundant sequence in that OTU. The RDP classifier analysis was used to assign 16S rRNA sequences to the phylum level's taxonomical hierarchy.

### RNA Extraction and RT-qPCR

Mice colon tissues were harvested and grinded by a precooled mortar with liquid nitrogen, and then total RNA was extracted from mice colon tissues using TRIzol Reagent (Takara, Dalian, China). One Step PrimeScript RT reagent Kit (Takara, Dalian, China, RR037A) was used for complementary DNA synthesis. Moreover, quantitative PCR amplification was performed using the SYBR Select Master Mix (Roche, 04913914001). For each sample, 100 ng of cDNA template was amplified in PCR reactions on a CFX96TM Real-Time System (Bio-Rad, United States). The quantitative PCR reaction conditions included an initial denaturation step at 95°C for 5 min, followed by 40 cycles at 95°C for 15 S, 60°C for 30 S, and 72°C for 20 S. The mRNA expression levels were normalized to β-actin as a housekeeping gene, and the data were calculated using the comparative Ct method 2^−ΔΔCt^ and expressed as fold change compared with the corresponding control. Primer sequences were designed and showed in Table 2.

**TABLE 2:** Colon tissues Real-time PCR primers.

Target	Primer sequence
COX-2	Forward -5′-CCG​TGG​GGA​ATG​TAT​GAG​CA-3′
	Reverse -5′-ATC​CAG​TCC​GGG​TAC​AGT​CA-3′
Muc2	Forward - 5′-AAC​GAT​GCC​TAC​ACC​AAG​GTC-3′
	Reverse - 5′-ACT​GAA​CTG​TAT​GCC​TTC​CTC​A-3′
β-actin	Forward -5′-GGC​GGA​CTG​TTA​CTG​AGC​TG-3′
	Reverse -5′-CTG​CGC​AAG​TTA​GGT​TTT​GTC​A-3′

Furthermore, the total DNA of the fecal samples was extracted with TIANamp Stool DNA Kit (DP328). The relative content of *Bacteroides fragilis, Lactobacillus, Bacillus coagulans, Sulphate-reducing bacteria (SRB), Akkermansia muciniphila,* and *Bifidobacterium* was examined with RT-PCR on CFX96TM Real-Time System. Primer sequences were designed and showed in Table 3.

**TABLE 3:** Bacteria Real-time PCR primers.

Target	Primer sequence (5′-3′)	Anneal Tm (°C)
*Bacteroides fragilis Lactobacillus*	F-ATAGCCTTTCGAAAGRAAGAT R-ATAGCCTTTCGAAAGRAAGAT F-GCAGCAGTAGGGAATCTTCCA R-GCATTYCACCGCTACACATG	50 60
*Bacillus coagulans*	F-GGTGGTAAATACTACGGTAATGGGGT R-GTGTCTAAATTACTGGTTGATTCGT	58
*Sulfate-reducing bacteria*	F-TGGCAGATMATGATYMACGGG R-GGGCCGTAACCGTCCTTGAA	60
*Akkermansia muciniphila*	F-CCTTGCGGTTGGCTTCAGAT R-CAGCACGTGAAGGTGGGGAC	55
*Bifidobacterium*	F-GCCACATTGGGACTGAGATA R-CCTACGAGCCCTTTACGC	60
All bacteria	F-TCCTACGGGAGGCAGCAGT R-GGACTACCAGGGTATCTATCCTGTT	58

### Statistics Analysis

All experimental results were presented as the mean ± SEM from at least three independent experiments. The Statistical differences among groups were tested by one-way analysis of variance (ANOVA). All Statistics analyses were performed using GraphPad Prism 8.0.2 and differences were considered significant at *p* < 0.05 (*p < 0.05, **p < 0.01).

## Results

### The Primary Components of SND

Four typical peaks of Quercetin, Ammonium Glycyrrhizinate, 6-Gingerol, and Carbenoxolone were determined by comparing the reference substance(Figures 1B,C). The reference substance peaks are shown in Supplementary Figure S1. Fingerprint similarity of 10 batches of SND is shown in Supplementary Table S1.

### The Effect of SND on CRC

**FIGURE 2:**
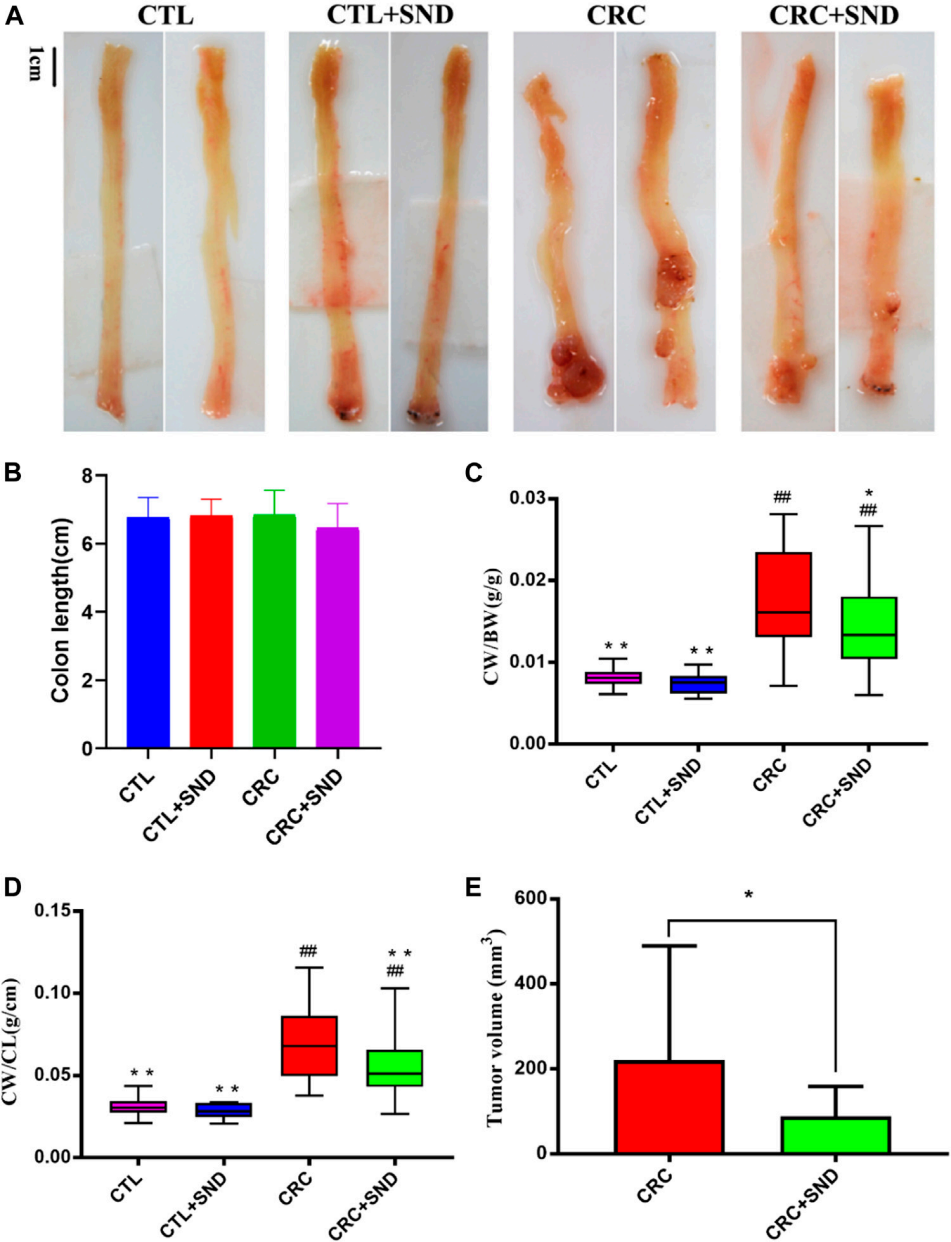
The effect of SND on colonic phenotype. **(A)** Colonic phenotype of different groups, *n* = 5. **(B)** Colon length of different groups, *n* = 5. **(C,D)** Colon weight (CW)/Body weight (BW) and colon weight (CW)/Body weight (BW) are chosen to be indicators of colon weight, *n* = 5. **(E)** Tumor volume of different groups, *n* = 5. Compared to CRC group, *p < 0.05, **p < 0.01; compared to CTL group, ##*p* < 0.01.

After 3 DSS cycles, the colorectal cancer model was successfully established, and the tumorigenic rate of the mouse tumor model is 100%. Consistent with previous reports (Thaker et al., 2012), a single intraperitoneal injection of the carcinogen AOM, followed by 3 rounds of 2.5% DSS intake induced the development of multiple tumors in the middle to the distal colon of mice. According to the pathological observation, tumors in the CRC group were mostly moderate dysplasia or severe dysplasia; however, tumors in the CRC + SND group were mild dysplasia. The CRC + SND group tumors were significantly less and smaller compared to the CRC group (Figure 2A). The colon weight and the tumor volume were selected to measure the degree of colorectal cancer severity. As shown in (Figures 2C–E), the colon with the tumor was more weight than that without, and the colon treated with SND was lighter than the CRC group.

**FIGURE 3:**
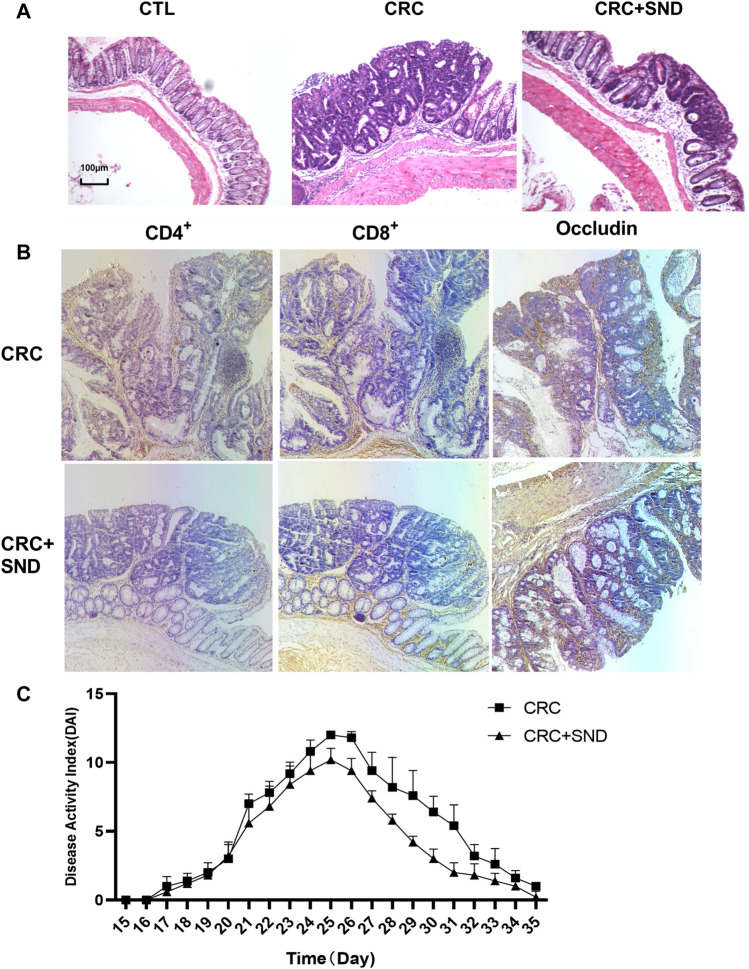
Pathological and immunohistochemical analysis. **(A)** The pathological phenotype of different groups. SND can Reduce the degree of tumor malignancy. **(B)** Immunohistochemical results of CD4^+^, CD8^+^ T cells, and Occludin quantity in CRC and CRC + SND group. **(C)** The disease Activity Index (DAI) of CRC and CRC + SND groups in the acute enteritis phase (Day15-Day35).

On the other hand, we performed a DAI score during the acute enteritis phase (day15–35). The results demonstrated that the SND group performed better at assessing the severity of enteritis or the recovery rate (*p* < 0.0001, Figure 3C).

### The Protective Effect of SND on Colonic Epithelium

The CTL and CTL + SND group's colonic crypts were arranged neatly; mucosal and muscular layer structure was complete. The CRC group mice colon mucosa was partially damaged; crypt grew separately and irregularly companied with glands. On the other hand, the CRC + SND group mice colon was slightly protected (Figure 3A).

The CD8^+^ T cells in CRC + SND group mice's colon tumors were more than the CRC group. On the contrary, there were fewer CD4^+^ T cells in the CRC + SND group than the CRC group. Furthermore, the CRC + SND group's Occludin expression was also slightly more than the CRC group (Figure 3B). SND reduced the mRNA expression levels of typical biomarkers in CRC.

**FIGURE 4:**
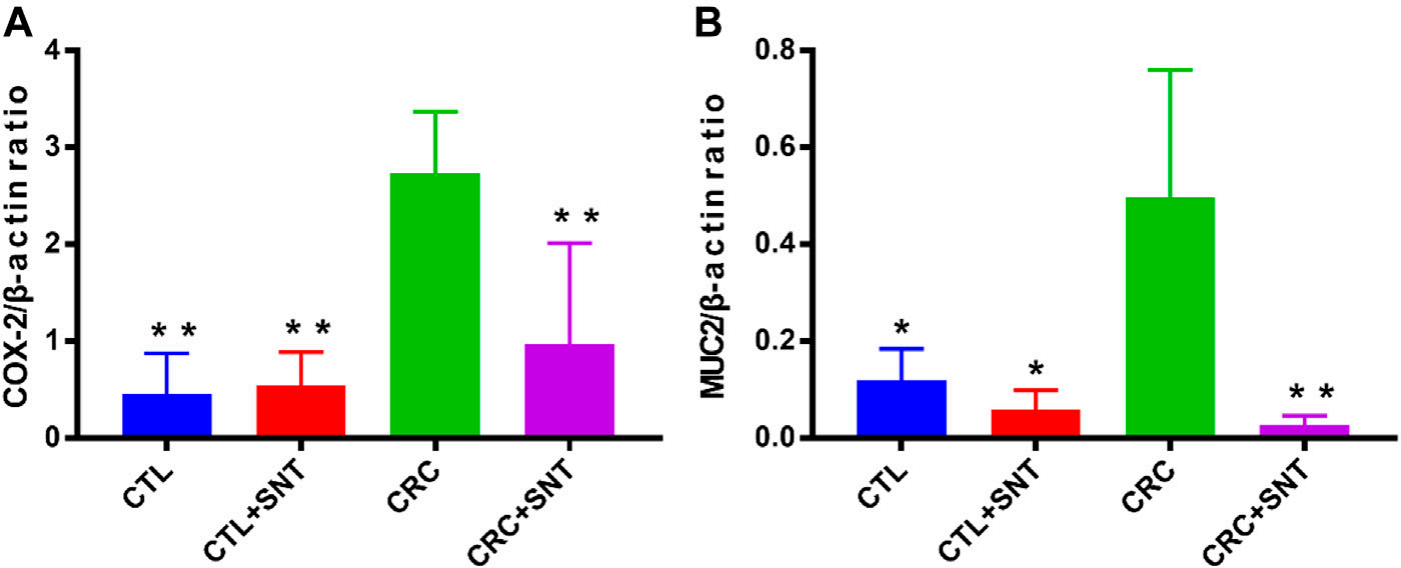
Changes of biomarkers in mouse CRC. **(A)** Relative mRNA expression of COX-2, *n* = 3. **(B)** Relative mRNA expression of MUC2, *n* = 3. Compared to CRC group, *p < 0.05, **p < 0.01.

Both Cyclooxygenase 2(COX-2) and Mucin-2(MUC2) were specific biomarkers for CRC (Melis et al., 2010; Ayiomamitis et al., 2019). As an inducible enzyme, COX-2 can be rapidly synthesized when cells were stimulated. However, researches have shown that COX-2 was not expressed at rest and is upregulated in various cancers, including colorectal cancer, and its specific inhibitor can inhibit tumor cell growth (Ayiomamitis et al., 2019). Besides, MUC2 was downregulated in general colorectal cancer and highly expressed in mucinous adenocarcinoma (Melis et al., 2010). The RT-qPCR results illustrated that both COX-2 and MUC2 were highly expressed in the CRC group but decreased in the CRC + SND group (Figure 4).

### 4SND Treatment Regulated Expression Levels of Cytokines in CRC

**FIGURE 5:**
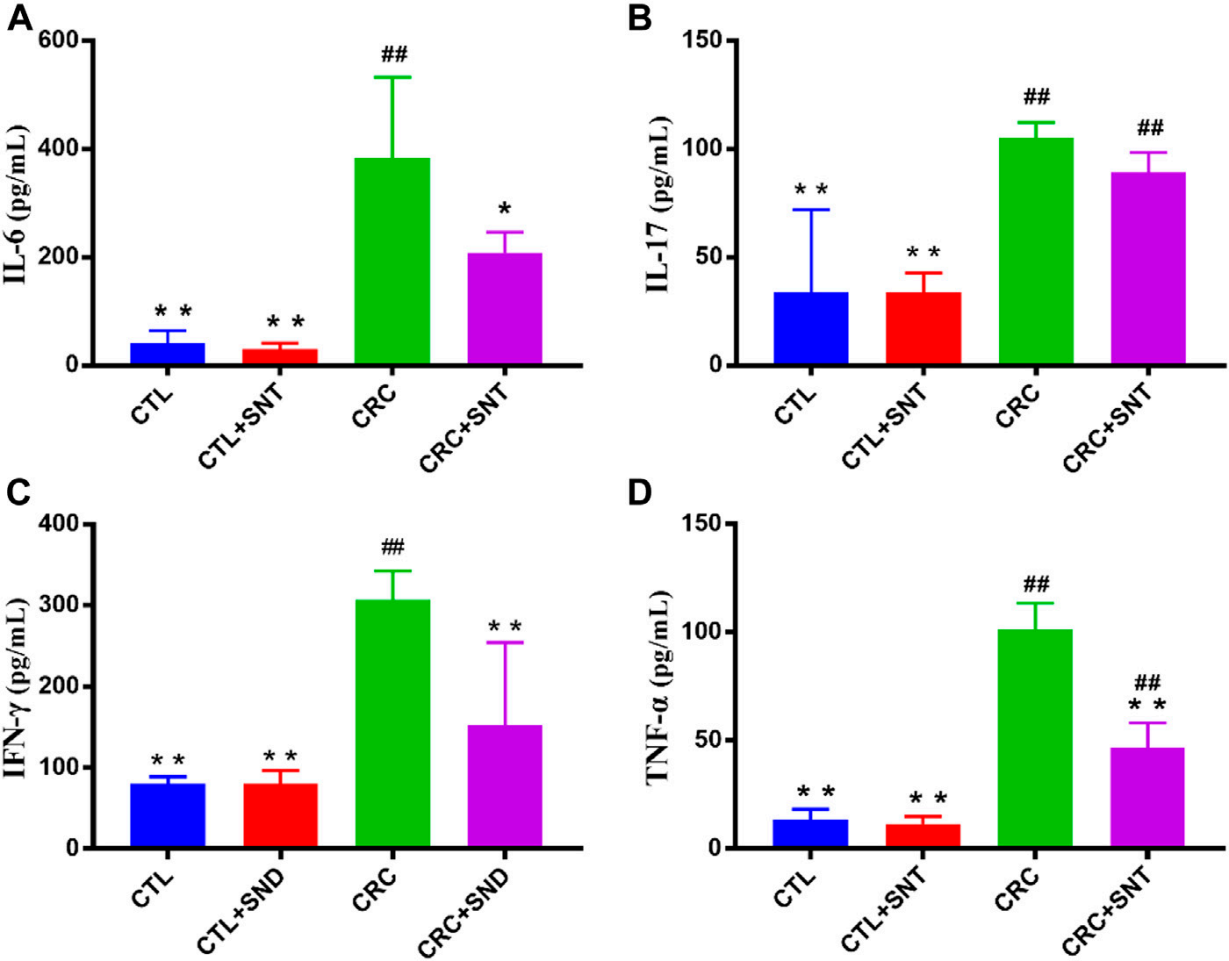
Changes of proinflammatory cytokines in mouse colon. **(A)** IL-6 expression in different groups, *n* = 3. **(B)** IL-17 expression in different groups, *n* = 3. **(C)** IFN-γ expression in different groups, *n* = 3. **(D)** TNF-α expression in different groups, *n* = 3. Compared to CRC group, *p < 0.05, **p < 0.01; compared to CTL group, ##*p* < 0.01.

In our research, the results indicated that CRC group mice colon had a higher expression level of IL-6, IL-17, and TNF-α compared to the CTL group. In comparison, CRC + SND group mice had less expression of IL-6, IL-17, and TNF-α compared to the CRC group (Figures 5A,B,D). Thus, SND can alleviate the CRC by regulating cytokines' expression levels, including IL-6, IL-17, and TNF-α. Also, SND can downregulate IFN-γ in the colon and decrease the immune response to flora, reducing intestinal inflammation(Figure 5C).

### SND Treatment Changed the Relative Abundance of Intestinal Microbiota at Different Taxonomic Levels

**FIGURE 6:**
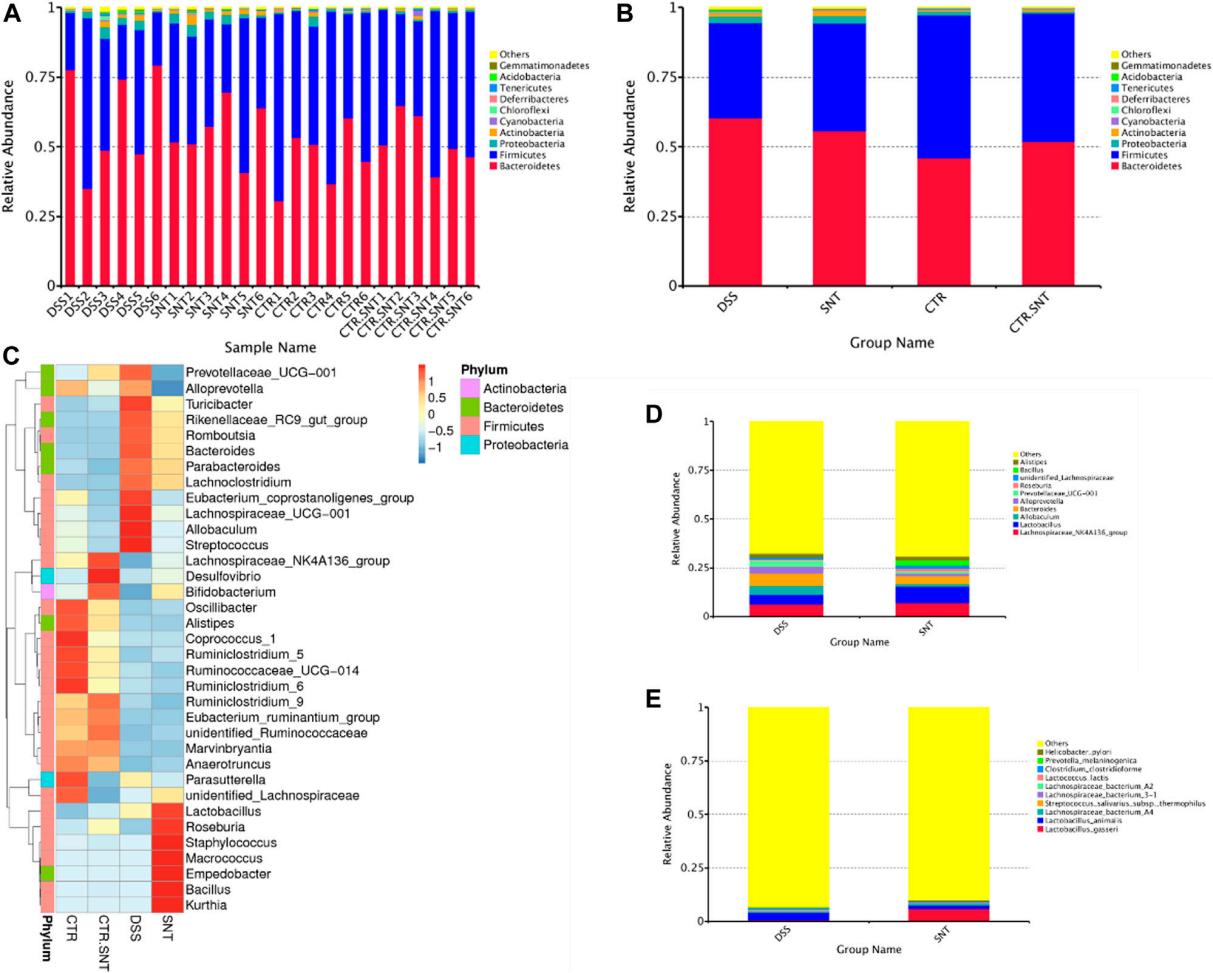
The relative abundance of mouse intestinal microbiota. **(A,B)** The Relative abundance display of phylum-level species. **(C)** Phylum-level species abundance clustering heatmap. **(D)** Relative abundance display of genus-level species. **(E)** The relative abundance of species. Group information in Figure: CTR-CTL, DSS-CRC, SNT-CRC + SND, CTR.SNT-CTL + SND.

The top 10 species were listed as followed in abundance at the phylum level for each sample in different groups: *Bacteroidetes, Firmicutes, Proteobacteria, Actinobacteria, Cyanobacteria, Chloroflexi, Deferribacteres, Tenericutes, Acidobacteria, Gemmatimonadetes,* and *Others.* Among them, *Bacteroidetes* took the highest proportion in the CRC group, less in the CTL and CRC + SND group; *Firmicutes* took the highest proportion in the CTL group and took the lowest ratio in the CRC group. However, our results showed that *Firmicutes* were increased in the CRC + SND group after being treated with SND (Figures 6A,B). *Roseburia,* a typical bacteria producing short-chain fatty acid (SCFAs), and *Lactobacillus* assembled in the CRC + SND group. Moreover, SND increased probiotics' content, including *_NK4A136_group and Bifidobacterium,* in disease-free mice(Figures 6C–E).

### SND Treatment Modulated the Gut Microbial Composition in CRC

**FIGURE 7:**
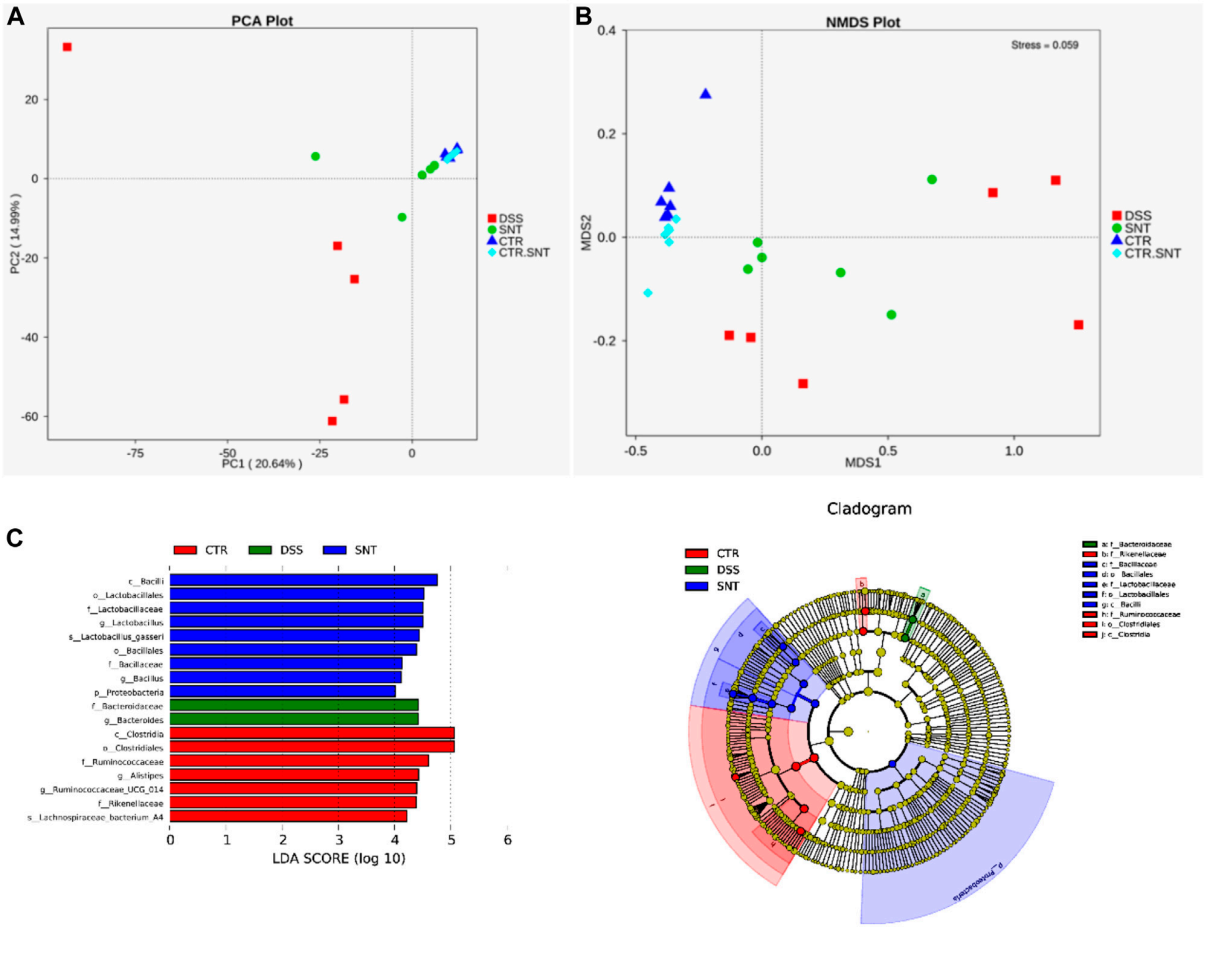
Diversity analysis of gut microbiota in mice. **(A)** PCA analysis. **(B)** NMDS analysis. **(C)** LEfSe analysis. *Bcteroidaese*, *Bacteroides* in the CRC group and *Lactobacillales*, *Lactobacillaceae*, *Bacillales*, *Bacillaceae* in the CRC + SND group, which made a big difference between groups by using LEfSe analysis were screened.

NMDS and PCA analysis showed that the CTL and CTL + SND group's gut microbiota structure was similar, and there was no apparent difference between them. The CRC group and the CRC + SND group's gut microbiota significantly differ from the CTL group, and the difference between or within these two groups was distinct. This result might suggest that SND can regulate the microbiota structure in mice and make it return to a reasonable level (Figures 7A,B). We also screened out dominant bacteria like *Bacteroides* in the CRC group and *Lactobacillus*, *Bacillus* in the CRC + SND group using LEfSe analysis (Figures 7C,D). Furthermore, *Lactobacillus gasseri* and *Bacillus coagulans*, which are specific probiotics, get enriched in the CRC + SND group. *Bacteroides fragilis,* which is a typical harmful bacterium, get increased in the CRC group.

### SND Treatment Modulated the Pattern of Gut Microbial Community

In this case, 3 kinds of bacteria associated with CRC (Zhao et al., 2017) were screened out by 16SV4 region amplicon sequencing technology. They were *Bacteroides fragilis* enriched in the CRC group, *Lactobacillus* and *Bacillus coagulans* gathered in the SND group. The quantity of these 3 kinds of bacteria was tested using RT-qPCR, and the test results were consistent with the 16s rRNA sequencing analysis. Moreover, we still examined the quantity of *Sulphate-reducing bacteria*, *Akkermansia muciniphila*, and *Bifidobacterium*, which also made an essential impact on CRC (Li et al., 2016). *Bacteroides fragilis, Lactobacillus, Bacillus coagulans,* and another 3 critical species, including *Sulphate-reducing bacteria, Akkermansia muciniphila,* and *Bifidobacterium,* were examined by using RT-PCR. Their relative quantity was calculated in 2^−ΔΔCt^. *Bacteroides fragilis* and *Sulphate-reducing bacteria,* which were harmful to gut health, had a high expression level in the CRC group but less in the CRC + SND group.

**FIGURE 8:**
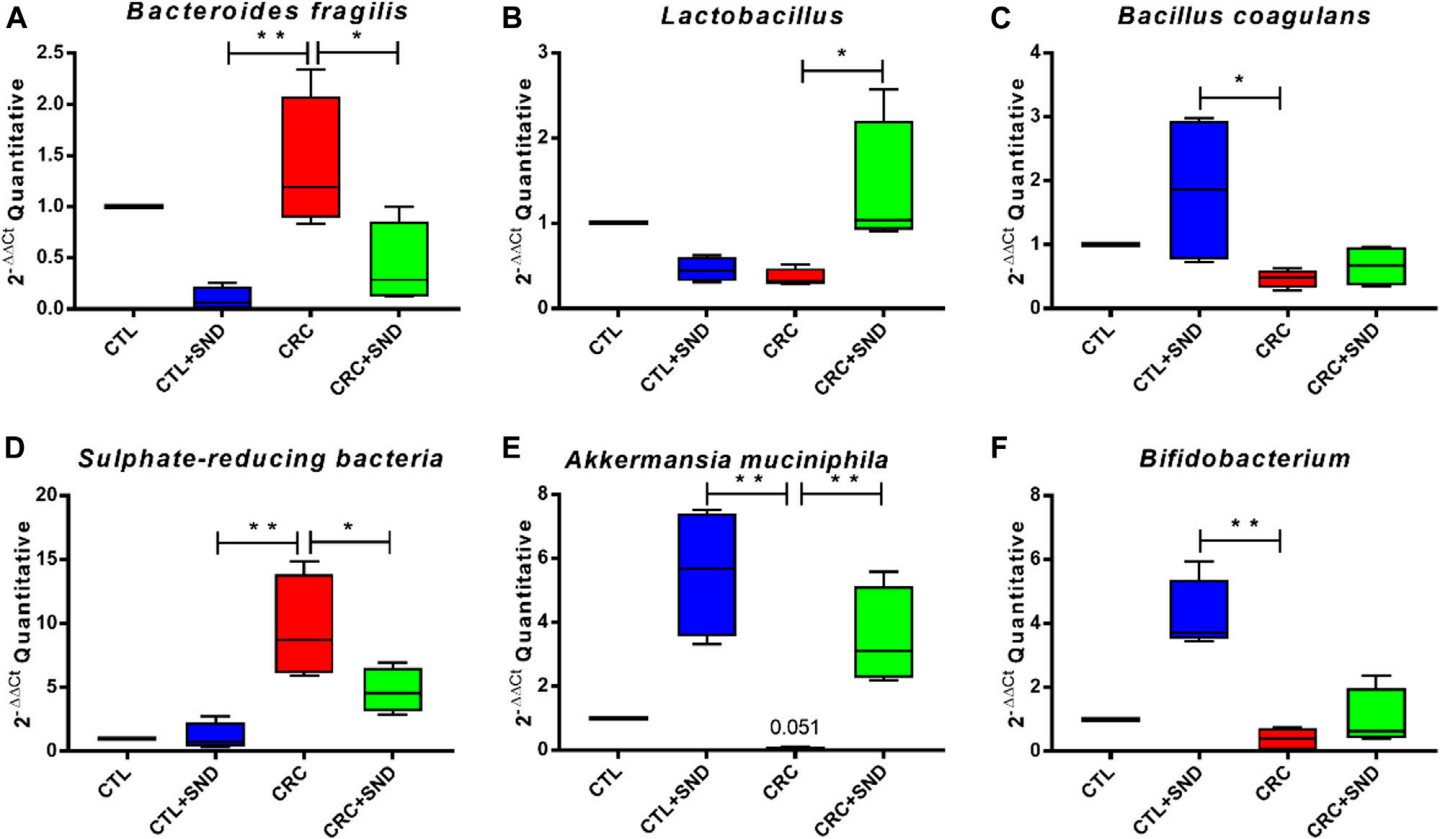
RT-qPCR examination for the screened species. **(A)** The relative abundance of *Bacteroides fragilis* in different groups. **(B)** The relative abundance of *Lactobacillus* in different groups. **(C)** The relative abundance of *Bacillus coagulans* in different groups. **(D)** The relative abundance of *Sulphate-reducing bacteria* in different groups. **(E)** The relative abundance of *Akkermansia muciniphila* in different groups. **(F)** The relative abundance of *Bifidobacterium* in different groups. Compared to CRC group, *p < 0.05, **p < 0.01.

On the contrary, *Bacillus coagulans, Lactobacillus, Akkermansia muciniphila,* and *Bifidobacterium* could improve gut health (Bultman, 2017) were raised in the CRC + SND group. Moreover, after the SND treatment, *Bacteroides fragilis* was significantly decreased, and *Bacillus coagulans, Akkermansia muciniphila, Bifidobacterium* were increased in mice. These results suggested that SND could increase the number of beneficial bacteria and reduce the harmful ones (Figure 8), which further confirmed that SND could ameliorate CRC development by regulating the intestinal microbial structure pattern community.

## Discussion

SND is an oral compound decoction. Although there have been cases of treating intestinal inflammatory diseases in TCM clinics, the SND drug's effect is still unclear. This research successfully constructed an AOM/DSS-induced mouse colorectal cancer model to promote its application. Moreover, as our results showed, the colonic phenotype results suggested that SND can alleviate mice colorectal cancer.

The main active ingredients we detected in SND were Quercetin, 6-Gingerol, Ammonium glycyrrhizate, and Carbenoxolone. Quercetin can inhibit CRC cell proliferation by decreasing NF-κB and MAPK pathways(Darband et al., 2018). 6-Gingerol can inhibit CRC development through inhibiting β-catenin, PKCε, and GSK-3β pathways (Lee et al., 2008). And Carbenoxolone was proved to inhibit colon cancer cells' growth by inhibiting pannexin-1 (Alhouayek et al., 2019). However, these are not all the useful substances in SND. It is just a superfluous exploration. In addition to these, SND also contains about 200 active substances such as aconitine and hypaconitine (Chen et al., 2014). Current chemomics and pharmacology proved that the total alkaloids, total gingerols, total flavones(polyphenol), and total saponins are the primary active ingredients in SND (Yue et al., 2009; Zhang and Ye, 2009; Chen et al., 2013). Most of them are potential anti-inflammatory substances, which have the ability to regulate immunity and metabolism (Chen et al., 2016). Although clarifying the effects of individual active ingredients is a way to explain the mechanism of SND alleviating CRC, the impact of TCM is not only the sum of several active ingredients. According to the TCM theory, Fuzi and Ganjiang can enhance the body's yang qi. The quantity of yang qi is related to the state of life and health. When the amount of yang qi in the body is too small, it will lead to various diseases, including low gastrointestinal temperature, limb ulceration, and even death. The primary function of SND in TCM theory is to restore yang qi and recover the critical condition of the dying patient, improving body temperature (Min and Guining, 2018). Practitioners of TCM believe that the low temperature of the gastrointestinal tract causes gastrointestinal diseases. Therefore, in modern medical applications, SND is also used to treat diarrhea, acute gastroenteritis, and related gastrointestinal cancer (Hua, 2019; Xian-tao, 2019).

Nevertheless, SND is still proven by us to suppress CRC through immune regulation. We found that SND has an inhibitory effect on inflammatory factors. Many studies have demonstrated that Th2 and Th17 cells and the cytokines secreted from them(TNF-α, IL-6, and IL-17) play a vital role in CRC's onset and development (Chen et al., 2017). IL-6 was considered to be a multifunctional NF-κB-regulated cytokine that acts on epithelial and immune cells. The proliferative and survival effects of IL-6 were mediated mainly by the transcription factor STAT3, whose intestinal epithelial cells (IEC)-specific ablation profoundly impacted CAC tumorigenesis (Matsumoto et al., 2010). Meanwhile, the NF-κB-IL-6-STAT3 cascade has been demonstrated as an essential regulator of the proliferation and survival of tumor-initiating IECs (Grivennikov et al., 2009). Moreover, IL-17 was a cytokine that bridges the adaptive and innate immune systems (Omrane et al., 2014). IL-17 is produced by CD4^+^ T helper cells named Th17 cells controlled by transcription factor retinoic-acid receptor-related orphan receptor (ROR)γt (Ivanov et al., 2006). In mice, naïve T cells can differentiate into Th17 cells producing IL-17A cytokine in the presence of IL-6 (Francescone et al., 2016). Blockade of IL-17A was ineffective and even exacerbated IBD in some patients due to the critical role IL-17A plays in maintaining epithelial barrier homeostasis (Higgins et al., 2016). Conversely, in colitis-associated cancer(CAC) and spontaneous CRC, the expression of IL-17A was discovered to be elevated, which led to a worsening disease progression (Higgins et al., 2016). Additionally, the essential involvement of a transcription factor, NF-κB, in this colon carcinogenesis model (Greten et al., 2004) prompted us to investigate the intracolonic expression of TNF-α as it is a potent activator of NF-κB (Liu, 2005). Furthermore, there were reduced tumor incidence in TNF-Rp55(TNF-α′s primary receptor, p55)-deficient (TNF-Rp55^–/–^) mice (Popivanova et al., 2008). In this research, consistent with previous studies, we found that the CRC group mice colon had a higher expression level of IL-6, IL-17, TNF-α, and IFN-γ than the CTL group. However, these cytokines were downregulated in the SND group. This result suggests that SND can relieve CRC by downregulating Inflammatory factors in mice colon.

Immunohistochemistry analysis showed more CD8^+^ T cells and fewer CD4^+^ T cells in the SND group mice's colon than the CRC group. The increase of CD8^+^ T cells after SND treatment means that SND can mobilize the cellular immune system to execute tumor cytotoxicity. Moreover, from the cytokine changes mentioned above, the decreased CD4^+^ T cells in SND group mice hypothesize that SND could reduce Th2 and Th17 cells, thus restraining NF-κB-IL-6-STAT3 cascade reaction. These inferences will be investigated in the future.

Moreover, SND could increase the quantity of Occludin in the intestinal mucosa, significantly reduced in the CRC group. In this case, the mice's colorectal cancer induced by AOM/DSS was alleviated by SND by enhancing the crypt's tight junctions and protecting the intestinal barrier. If intestinal mucosa were damaged, the intestine would expose to microbiota and its production continuously. The immune system stimulated by microbiota would induce low-grade inflammation consecutively, increasing the risk of colorectal cancer. A healthy intestinal barrier can prevent lipopolysaccharide(LPS) and other infection sources from causing inflammation and loss of integrity of the intestinal wall (Zeng et al., 2019). These results indicate that SND can reduce inflammation via protecting the enteric epithelial barrier and reducing immune cells involved in inflammation during CRC treatment.

Increasing research has shown that gut microbiota played a decisive role in developing colorectal cancer (Bultman, 2017). Intestinal inflammation could not cause CRC without gut microbiota or bacterial production (Arthur et al., 2012). Wang T et al. found that *Bacteroides fragilis* was more enriched in CRC patients than healthy people (Wang et al., 2012), and it was proved could directly participate in the development of CRC (Sears, 2009). *Bacteroides fragilis* exists both as a part of healthy commensal flora and as pathogenic bacteria expressing a zinc metalloprotease called *Bacteroides fragilis* toxin (BFT). BFT causes a pronounced loss of ZO-1 localization at the tight junctions by 4 h, although effects can be seen as early as 2 h after treatment with the toxin (Hodges and Hecht, 2012). Inflammation caused by *Bacteroides fragilis* could induce Spermine oxidase in the intestine, which would damage DNA (Goodwin et al., 2011). Similarly, sulfurated hydrogen produced by *Sulphate-reducing bacteria* digesting food could also damage DNA (Louis et al., 2014). Furthermore, SND could downregulate these two kinds of bacteria to control the development of CRC.

On the other hand, some health-promoting microbes, including *Bacillus coagulans, Akkermansia muciniphila, Bifidobacterium, Lactobacillus gasseri,* were increased by SND. *Bifidobacterium* could inhibit *Bacteroides*' growth from preventing its damage to intestinal epithelial cells (Shiba et al., 2003). Sivan et al. reported a decrease in tumor size when treated with a *Bifidobacterium* cocktail(orally) alone. Moreover, using programmed cell death ligand-1 (PD-L1) blockade immunotherapy and *Bifidobacterium* cocktail together almost stopped the tumor outgrowth. *Bifidobacterium* treatment improved immune responses, including CD8^+^T-cells activation and costimulation, cytokine-cytokine receptor interaction, and augmented dendritic cell (DC) function, and the chemokine-mediated recruitment of immune cells to the tumor microenvironment (Sivan et al., 2015). *Lactobacillus* could generate antibacterial metabolites, regulating the immune defense and non-Immune defense (Ljungh and Wadström, 2006). L. gasseri strains showed an anti-inflammatory impact on HeLa cells by decreasing the production of TNF-α and increasing IL-10 production (Sungur et al., 2017). *Akkermansia muciniphila*, which can inhabit intestinal mucus, could improve inflammation, regulate the intestinal immune by degrading mucin and generating methyl acetic (Le Poul et al., 2003). Oral supplementation with *A. muciniphila* after FMT with nonresponder feces restored the efficacy of PD-1 blockade (Routy et al., 2018). *Bacillus coagulans* were proved to improve chronic colitis, enhance immunity, inhibit enteropathogenic bacteria, and repair the intestinal mucosa (Bomko et al., 2017). The treatment with *Bacillus coagulans* and the prebiotic inulin significantly inhibits serum amyloid A protein in arthritic rats and promotes a significant TNF release decrease (de Oliveira, 2019). Moreover, a polyphenol-rich diet was proved could reduce serum zonulin levels(an indirect marker of intestinal permeability) and blood pressure and increase fiber-fermenting and butyrate-producing bacteria (Del Bo’ et al., 2020). Therefore, the flavones, a kind of polyphenol, in SND may be one reason for the increase of these probiotics.

**FIGURE 9:**
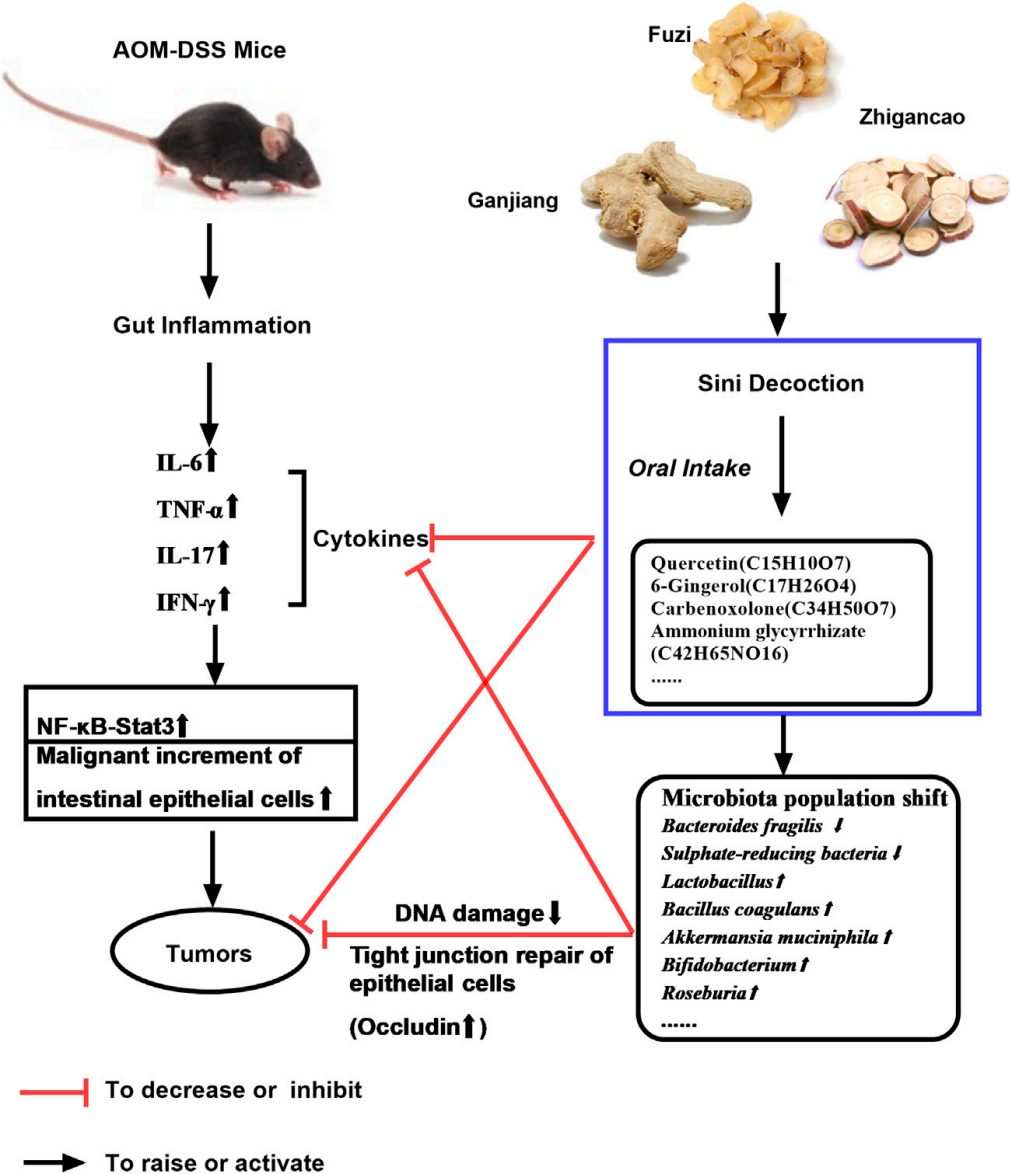
Schematic diagram of SND on AOM/DSS-induced colon cancer.

Furthermore, microorganisms can participate in body immunity through TLR-4 (Chen et al., 2017). At this level, the composition of mice gut microbiota, which was ameliorated by SND, can influence tumors' onset and development via regulating the immunity system. Probiotics, including *Bifidobacterium, Bacillus coagulans,* and *Lactobacillus* et., can downregulate TNF-α, IL-6, and IL-17, subsequently inhibiting the NF-κB/STAT3 pathway and malignant proliferation of mucosal epithelial cells. Moreover, they can protect the tight junction (Occludin, ZO-1) between colonic epithelial cells, prevent DNA damage, and reduce cancer risk. Interestingly, Chen Si et al. indicated that SND's active components could directly bind to TNF-α, inhibiting TNF-α-mediated NF-κB activation (Chen et al., 2016), which is consistent with our results. Therefore, SND can directly(binding) or indirectly (with microbiota) downregulate the NF-κ-B/STAT3, and decrease the DNA damage, subsequently restraining the formation and expansion of colon tumors. A schematic diagram of SND on AOM/DSS-induced colon cancer was provided (Figure 9). In future research, how SND gets an impact on the immune system and gut microbiota will still be our primary interest.

In this study, we found that SND downregulated the expression levels of COX-2 and MUC2. The decrease in these two tumor markers after oral SND treatment implies that SND can control CRC development. Also, SND inhibited the secretion of inflammatory cytokines; in the meantime, it can upregulate the expression of CD8^+^ T lymphocytes and Occludin in the colonic mucosal layer. These results indicated that SND could restrain CRC development by decreasing interrelated cytokines, protecting the intestinal barrier. Furthermore, we did not observe any side effects in animal experiments, which means that it is a safe medicine so far. Therefore, our findings suggested that SND can become a new adjuvant therapy for CRC, and this study may provide theoretical support for exploring the mechanism of SND affecting CRC. Moreover, SND extract was suggested could modulate the structure and relative abundance of the gut microbial community. However, temporarily, we have no evidence of a causal relationship between the ability of SND to alter gut microbiota and its ability to alleviate CRC. This will be the focus of our next work. And we will continue to explore how the body metabolizes Sini Decoction extract. We speculated that the shaping of intestinal flora by TCM could become a breakthrough point in the CRC treatment and the further elaboration of its drug action mechanism.

## Data Availability

The raw data supporting the conclusions of this article will be made available by the authors, without undue reservation.

## Abbreviations

CAM, Complementary and alternative medicine; SND, Sini Decoction; TCM, Traditional Chinese Medicine; CRC, Colorectal cancer; AOM, Azoxymethane; DSS, Dextran sulfate sodium; MUC2, Mucin-2; COX-2, Cyclooxygenase 2; IBD, Inflammatory bowel disease; PCA, Principal Component Analysis; PCoA, Principal Co-ordinates Analysis; LDA, Linear Discriminant Analysis.

## References

[B1] Adami H.O. Bretthauer M. Emilsson L. Hernán M. A. Kalager M. Ludvigsson J. F. (2016). The continuing uncertainty about cancer risk in inflammatory bowel disease. Gut. 65, 889–893. 10.1136/gutjnl-2015-311003 27008845 PMC5226357

[B2] Alhouayek M. Sorti R. Gilthorpe J. D. Fowler C. J. (2019). Role of pannexin-1 in the cellular uptake, release and hydrolysis of anandamide by T84 colon cancer cells. Sci. Rep. 9, 1–12. 10.1038/s41598-019-44057-x 31110238 PMC6527687

[B3] Arthur J. C. Perez-Chanona E. Mühlbauer M. Tomkovich S. Uronis J. M. Fan T.J. (2012). Intestinal inflammation targets cancer-inducing activity of the microbiota. Science 338, 120–123. 10.1126/science.1224820 22903521 PMC3645302

[B4] Ayiomamitis G. D. Notas G. Vasilakaki T. Tsavari A. Vederaki S. Theodosopoulos T. (2019). Understanding the interplay between COX-2 and hTERT in colorectal cancer using a multi-omics analysis. Cancers 11, 1536. 10.3390/cancers11101536 PMC6827032 31614548

[B5] Bomko T. V. Nosalskaya T. N. Kabluchko T. V. Lisnyak Y. V. Martynov A. V. (2017). Immunotropic aspect of the *Bacillus* coagulans probiotic action. J. Pharm. Pharmacol. 69, 1033–1040. 10.1111/jphp.12726 28397382

[B6] Bultman S. J. (2017). Interplay between diet, gut microbiota, epigenetic events, and colorectal cancer. Mol. Nutr. Food Res. 61, 1500902. 10.1002/mnfr.201500902 PMC5161716 27138454

[B7] Chen J. Pitmon E. Wang K. (2017). Microbiome, inflammation and colorectal cancer. Semin. Immunol. 32, 43–53. 10.1016/j.smim.2017.09.006 28982615

[B8] Chen S. Jiang H. Cao Y. Wang Y. Hu Z. Zhu Z. (2016). Drug target identification using network analysis: taking active components in Sini decoction as an example. Sci. Rep. 6, 24245. 10.1038/srep24245 27095146 PMC4837341

[B9] Chen S. Wu S. Li W. Chen X. Dong X. Tan G. (2014). Investigation of the therapeutic effectiveness of active components in Sini decoction by a comprehensive GC/LC-MS based metabolomics and network pharmacology approaches. Mol. Biosyst. 10, 3310–3321. 10.1039/c4mb00048j 25315049

[B10] Chen Y. L. Zhuang X. D. Xu Z. W. Lu L. H. Guo H. L. Wu W. K. (2013). Higenamine combined with [6]-gingerol suppresses doxorubicin-triggered oxidative stress and apoptosis in cardiomyocytes via upregulation of PI3K/akt pathway. Evidence-Based Complement. Altern. Med. 2013, 970490. 10.1155/2013/970490 PMC3687593 23861719

[B11] Choi C. H. R. Bakir I. A. Hart A. L. Graham T. A. (2017). Clonal evolution of colorectal cancer in IBD. Nat. Rev. Gastroenterol. Hepatol. 14, 218–229. 10.1038/nrgastro.2017.1 28174420

[B12] Darband S. G. Kaviani M. Yousefi B. Sadighparvar S. Pakdel F. G. Attari J. A. (2018). Quercetin: A functional dietary flavonoid with potential chemo-preventive properties in colorectal cancer. J. Cell. Physiol. 233, 6544–6560. 10.1002/jcp.26595 29663361 PMC13482535

[B13] de Oliveira G. L. V. (2019). “The gut microbiome in autoimmune diseases, in Microbiome and Metabolome in diagnosis, therapy, and other strategic applications. Amsterdam, Netherlands: Elsevier, 325–332. 10.1016/b978-0-12-815249-2.00033-6

[B14] Del Bo’ C. Bernardi S. Cherubini A. Porrini M. Gargari G. Hidalgo-Liberona N. (2020). A polyphenol-rich dietary pattern improves intestinal permeability, evaluated as serum zonulin levels, in older subjects: the MaPLE randomised controlled trial. Clin. Nutr. 10.1016/j.clnu.2020.12.014 33388204

[B16] Favoriti P. Carbone G. Greco M. Pirozzi F. Pirozzi R. E. M. Corcione F. (2016). Worldwide burden of colorectal cancer: a review. Updates Surg. 68, 7–11. 10.1007/s13304-016-0359-y 27067591

[B17] Francescone R. Hou V. Grivennikov S. I. Prevention C. Program C. Chase F. (2016). Cytokines, IBD, and colitis-associated cancer. Inflamm. Bowel Dis. 21, 409–418. 10.1097/MIB.0000000000000236.Cytokines PMC4481731 25563695

[B18] Goodwin A. C. Shields C. E. D. Wu S. Huso D. L. Wu X. Murray-Stewart T. R. (2011). Polyamine catabolism contributes to enterotoxigenic Bacteroides fragilis-induced colon tumorigenesis. Proc. Natl. Acad. Sci. 108, 15354–15359. 10.1073/pnas.1010203108 21876161 PMC3174648

[B19] Greten F. R. Eckmann L. Greten T. F. Park J. M. Li Z.-W. Egan L. J. (2004). IKKβ links inflammation and tumorigenesis in a mouse model of colitis-associated cancer. Cel. 118, 285–296. 10.1016/j.cell.2004.07.013 15294155

[B20] Grivennikov S. Karin E. Terzic J. Mucida D. Yu G.-Y. Vallabhapurapu S. (2009). IL-6 and Stat3 are required for survival of intestinal epithelial cells and development of colitis-associated cancer. Cancer Cel. 15, 103–113. 10.1016/j.ccr.2009.01.001 PMC2667107 19185845

[B21] Higgins P. D. R. Wehkamp J. Feagan B. G. Yao M. D. (2016). Secukinumab, a human anti-IL-17A monoclonal antibody, for moderate to severe Crohn’s disease: unexpected results of a randomised, double-blind placebo-controlled trial. Gut. 61, 1693–1700. 10.1136/gutjnl-2011-301668.Secukinumab PMC4902107 22595313

[B22] Hodges K. Hecht G. (2012). “Physiology of host-pathogen interactions,”in Physiology of the gastrointestinal tract. Amsterdam, Netherlands: Elsevier Inc., 2047–2073. 10.1016/B978-0-12-382026-6.00077-4

[B23] Hooper L. V. Littman D. R. Macpherson A. J. (2012). Interactions between the microbiota and the immune system. Science 336, 1268–1273. 10.1126/science.1223490 22674334 PMC4420145

[B24] Hua L. (2019). Sini decoction combined with acupuncture in treating 43 cases of pediatric acute enteritis. HENAN tradit. Chinese Med. 39, 1020–1023. 10.16367/j.issn.1003-5028.2019.07.0252

[B25] Ivanov I. I. Mckenzie B. S. Zhou L. Tadokoro C. E. Lepelley A. Lafaille J. J. (2006). The orphan nuclear receptor RORγt directs the differentiation program of proinflammatory IL-17+ T helper cells. Cel. 126, 1121–1133. 10.1016/j.cell.2006.07.035 16990136

[B26] Jian L. U. (2010). Study on Sini powders and different compatibilities in intervening experimental ulcerative colitis. Chin. J. Exp. Tradit. Med. Formulae 16, 9–12. 10.13422/j.cnki.syfjx.2010.16.043

[B27] Le Poul E. Loison C. Struyf S. Springael J.-Y. Lannoy V. Decobecq M.-E. (2003). Functional characterization of human receptors for short chain fatty acids and their role in polymorphonuclear cell activation. J. Biol. Chem. 278, 25481–25489. 10.1074/jbc.M301403200 12711604

[B28] Lee S. H. Cekanova M. Baek S. J. (2008). Multiple mechanisms are involved in 6-gingerol-induced cell growth arrest and apoptosis in human colorectal cancer cells. Mol. Carcinog. 47, 197–208. 10.1002/mc.20374 18058799 PMC2430145

[B29] Li J. Lin S. Vanhoutte P. M. Woo C. W. Xu A. (2016). Akkermansia muciniphila protects against atherosclerosis by preventing metabolic endotoxemia-induced inflammation in apoe −/− mice. Circulation 133, 2434–2446. 10.1161/CIRCULATIONAHA.115.019645 27143680

[B30] Li Z. (2018). Treatment of 40 cases of gastrointestinal dysfunction in colorectal cancer during rehabilitation with Xiaoyao Sanhe Wumei pill. Chin. Manip. Rehabil. Med. 9, 47–48. 10.19787/j.issn.1008-1879.2018.14.023

[B31] Liu Z. G. (2005). Molecular mechanism of TNF signaling and beyond. Cel. Res 15, 24–27. 10.1038/sj.cr.7290259 15686622

[B32] Ljungh A. Wadström T. (2006). Lactic acid bacteria as probiotics. Curr. Issues Intest. Microbiol. 7, 73–89. 16875422

[B33] Louis P. Hold G. L. Flint H. J. (2014). The gut microbiota, bacterial metabolites and colorectal cancer. Nat. Rev. Microbiol. 12, 661–672. 10.1038/nrmicro3344 25198138

[B34] Ma X. Hua J. Li Z. (2008). Probiotics improve high fat diet-induced hepatic steatosis and insulin resistance by increasing hepatic NKT cells. J. Hepatol. 49, 821–830. 10.1016/j.jhep.2008.05.025 18674841 PMC2588670

[B35] Matsumoto S. Hara T. Mitsuyama K. Yamamoto M. Tsuruta O. Sata M. (2010). Essential roles of IL-6 trans-signaling in colonic epithelial cells, induced by the IL-6/soluble–IL-6 receptor derived from lamina propria macrophages, on the development of colitis-associated premalignant cancer in a murine model. J. Immunol. 184, 1543–1551. 10.4049/jimmunol.0801217 20042582

[B36] Mattar M. C. Lough D. Pishvaian M. J. Charabaty A. (2011). Current management of inflammatory bowel disease and colorectal cancer. Gastrointest. Cancer Res. 4, 53–61. 21673876 PMC3109885

[B37] Melis M. Hernandez J. Siegel E. M. McLoughlin J. M. Ly Q. P. Nair R. M. (2010). Gene expression profiling of colorectal mucinous adenocarcinomas. Dis. Colon Rectum 53, 936–943. 10.1007/DCR.0b013e3181d320c4 20485009

[B38] Min L. Guining W. (2018). Shang Han Lun.

[B39] O’Hara A. M. Shanahan F. (2006). The gut flora as a forgotten organ. Embo Rep. 7, 688–693. 10.1038/sj.embor.7400731 16819463 PMC1500832

[B40] Omrane I. Baroudi O. Bougatef K. Mezlini A. Abidi A. Medimegh I. (2014). Significant association between IL23R and IL17F polymorphisms and clinical features of colorectal cancer. Immunol. Lett. 158, 189–194. 10.1016/j.imlet.2014.01.002 24440568

[B41] Popivanova B. K. Kitamura K. Wu Y. Kondo T. Kagaya T. Kaneko S. (2008). Blocking TNF-alpha in mice reduces colorectal carcinogenesis associated with chronic colitis. J. Clin. Invest. 118, 560. 10.1172/JCI32453DS1 18219394 PMC2213370

[B42] Rahman H. Kim M. Leung G. Green J. A. Katz S. (2017). Drug-herb interactions in the elderly patient with IBD: A growing concern. Curr. Treat. Options. Gastro. 15, 618–636. 10.1007/s11938-017-0154-y 28918484

[B43] Routy B. Le Chatelier E. Derosa L. Duong C. P. M. Alou M. T. Daillère R. (2018). Gut microbiome influences efficacy of PD-1-based immunotherapy against epithelial tumors. Science 359, 91–97. 10.1126/science.aan3706 29097494

[B44] Sears C. L. (2009). Enterotoxigenic Bacteroides fragilis: A rogue among symbiotes. Cmr. 22, 349–369. 10.1128/CMR.00053-08 PMC2668231 19366918

[B45] Shiba T. Aiba Y. Ishikawa H. Ushiyama A. Takagi A. Mine T. (2003). The suppressive effect of bifidobacteria onBacteroides vulgatus, a putative pathogenic microbe in inflammatory bowel disease. Microbiol. Immunol. 47, 371–378. 10.1111/j.1348-0421.2003.tb03368.x 12906096

[B46] Sivan A. Corrales L. Hubert N. Williams J. B. Aquino-Michaels K. Earley Z. M. (2015). Commensal Bifidobacterium promotes antitumor immunity and facilitates anti-PD-L1 efficacy. Science 350, 1084–1089. 10.1126/science.aac4255 26541606 PMC4873287

[B47] Sungur T. Aslim B. Karaaslan C. Aktas B. (2017). Impact of Exopolysaccharides (EPSs) of Lactobacillus gasseri strains isolated from human vagina on cervical tumor cells (HeLa). Anaerobe., 47, 137. 10.1016/j.anaerobe.2017.05.013 28554813

[B48] Tan G. Liu M. Dong X. Wu S. Fan L. Qiao Y. (2014). A strategy for rapid analysis of xenobiotic metabolome of Sini decoction *in vivo* using ultra-performance liquid chromatography-electrospray ionization quadrupole-time-of-flight mass spectrometry combined with pattern recognition approach. J. Pharm. Biomed. Anal. 96, 187–196. 10.1016/j.jpba.2014.03.028 24759592

[B49] Thaker A. I. Shaker A. Rao M. S. Ciorba M. A. (2012). Modeling colitis-associated cancer with azoxymethane (AOM) and dextran sulfate sodium (DSS). J Vis Exp. 11, 4100. 10.3791/4100 PMC3490277 22990604

[B50] Tong X. Xu J. Lian F. Yu X. Zhao Y. Xu L. (2018). Structural alteration of gut microbiota during the amelioration of human type 2 diabetes with hyperlipidemia by metformin and a traditional Chinese herbal formula: a multicenter, randomized, open label clinical trial. mBio 9, e02392-17. 10.1128/mBio.02392-17 29789365 PMC5964358

[B51] Wang T. Cai G. Qiu Y. Fei N. Zhang M. Pang X. (2012). Structural segregation of gut microbiota between colorectal cancer patients and healthy volunteers. ISME J. 6, 320–329. 10.1038/ismej.2011.109 21850056 PMC3260502

[B52] Wang W. Chen Q. Yang X. Wu J. Huang F. (2020). Sini decoction ameliorates interrelated lung injury in septic mice by modulating the composition of gut microbiota. Microb. Pathogenesis 140, 103956. 10.1016/j.micpath.2019.103956 31891794

[B53] Wu S. Rhee K.-J. Albesiano E. Rabizadeh S. Wu X. Yen H.-R. (2009). A human colonic commensal promotes colon tumorigenesis via activation of T helper type 17 T cell responses. Nat. Med. 15, 1016–1022. 10.1038/nm.2015 19701202 PMC3034219

[B54] Xian-tao S. (2019). Discussion on Jiawei Sini decoction in treating chronic enteritis. Guid. China Med. 17, 4–5. 10.15912/j.cnki.gocm.2019.11.004

[B55] Yang J. Yu J. (2018). The association of diet, gut microbiota and colorectal cancer: what we eat may imply what we get. Protein Cel. 9, 474–487. 10.1007/s13238-018-0543-6 PMC5960467 29713943

[B56] Yu Y. Li X. Wu B. Yao F. Zhang H. Li S. (2015). Therapeutic effect of Jiawei Huangqi decoction on prevention of delayed diarrhea caused by irinotecan chemotherapy in patients with advanced intestinal cancer. Beijing J. Tradit. Chin. Med. 6, 5–8. 10.16025/j.1674-1307.2015.06.001

[B57] Yue H. Pi Z. Song F. Liu Z. Cai Z. Liu S. (2009). Studies on the aconitine-type alkaloids in the roots of Aconitum Carmichaeli Debx. by HPLC/ESIMS/MSn. Talanta 77, 1800–1807. 10.1016/j.talanta.2008.10.022 19159802

[B58] Yue S. J. Wang W. X. Yu J. G. Chen Y. Y. Shi X. Q. Yan D. (2019). Gut microbiota modulation with traditional Chinese medicine: a system biology-driven approach. Pharmacol. Res. 148, 104453. 10.1016/j.phrs.2019.104453 31541688

[B59] Zeng Y. Zhang H. Zong L. Tsao R. Arie H. Izumo T. (2019). Lactobacillus pentosus S-PT84 prevents LPS-induced low-grade chronic inflammation in a C57BL/6J mouse model. J. Funct. Foods 62, 103526. 10.1016/j.jff.2019.103526

[B60] Zhang L. Song Y. Ma J. Wang Y. Zhang Y. Fan X. (2020). Sini decoction alleviates ulcerative colitis induced by dextran sodium sulfate in mice. J. Hainan Trop. Ocean Univ. 27, 92–98. 10.13307/j.issn.2096-3122.2020.02.14

[B61] Zhang Q. Ye M. (2009). Chemical analysis of the Chinese herbal medicine Gan-Cao (licorice). J. Chromatogr. A 1216, 1954–1969. 10.1016/j.chroma.2008.07.072 18703197

[B62] Zhao L. Zhang X. Zuo T. Yu J. (2017). The composition of colonic commensal bacteria according to anatomical localization in colorectal cancer. Engineering 3, 90–97. 10.1016/j.eng.2017.01.012

