# Supplementary material for: Sini Decoction Ameliorates Colorectal Cancer and Modulates the Composition of Gut Microbiota in Mice
Source: Front Pharmacol. 2021 Mar 11;12:609992. doi: 10.3389/fphar.2021.609992 (PMC7991589; doi:10.3389/fphar.2021.609992)
Supplement: Supplementary file 1 [file datasheet1.docx]

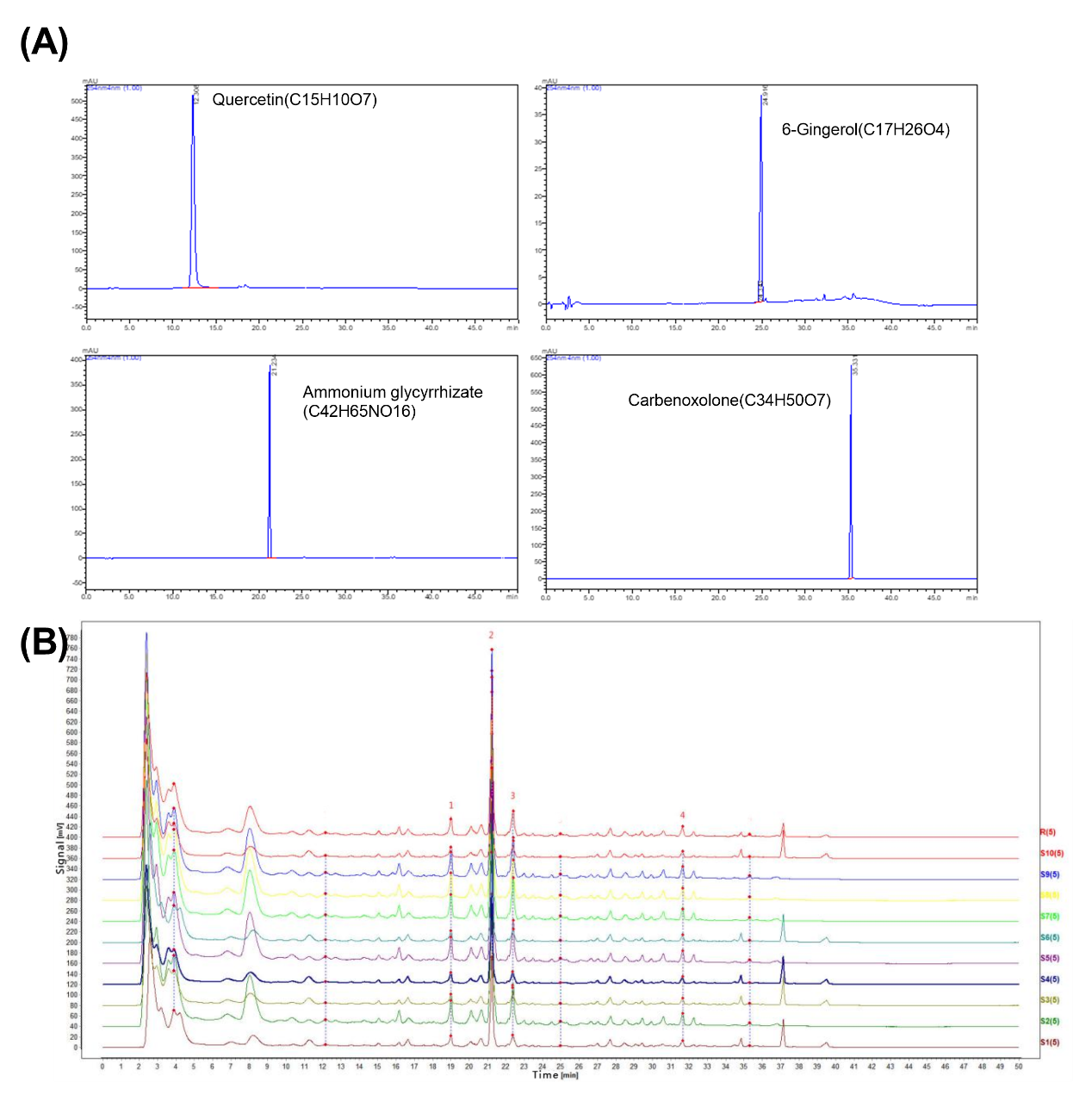


Figure 1. HPLC analysis results. (A) HQD extracts and purified chemical reference substances were used for quantitation by HPLC. (B) HPLC analysis fingerprint.

|  | S1 | S2 | S3 | S4 | S5 | S6 | S7 | S8 | S9 | S10 | reference  fingerprint |
| --- | --- | --- | --- | --- | --- | --- | --- | --- | --- | --- | --- |
| S1 | 1 | 0.928 | 0.798 | 0.798 | 0.73 | 0.999 | 0.928 | 0.928 | 0.928 | 0.798 | 0.915 |
| S2 | 0.928 | 1 | 0.965 | 0.965 | 0.932 | 0.928 | 0.999 | 0.999 | 0.999 | 0.965 | 0.999 |
| S3 | 0.798 | 0.965 | 1 | 0.999 | 0.994 | 0.798 | 0.965 | 0.965 | 0.965 | 0.999 | 0.973 |
| S4 | 0.798 | 0.965 | 0.999 | 1 | 0.994 | 0.798 | 0.965 | 0.965 | 0.965 | 0.999 | 0.973 |
| S5 | 0.73 | 0.932 | 0.994 | 0.994 | 1 | 0.73 | 0.932 | 0.932 | 0.932 | 0.994 | 0.944 |
| S6 | 0.999 | 0.928 | 0.798 | 0.798 | 0.73 | 1 | 0.928 | 0.928 | 0.928 | 0.798 | 0.915 |
| S7 | 0.928 | 0.999 | 0.965 | 0.965 | 0.932 | 0.928 | 1 | 0.999 | 0.999 | 0.965 | 0.999 |
| S8 | 0.928 | 0.999 | 0.965 | 0.965 | 0.932 | 0.928 | 0.999 | 1 | 0.999 | 0.965 | 0.999 |
| S9 | 0.928 | 0.999 | 0.965 | 0.965 | 0.932 | 0.928 | 0.999 | 0.999 | 1 | 0.965 | 0.999 |
| S10 | 0.798 | 0.965 | 0.999 | 0.999 | 0.994 | 0.798 | 0.965 | 0.965 | 0.965 | 1 | 0.973 |
| reference  fingerprint | 0.915 | 0.999 | 0.973 | 0.973 | 0.944 | 0.915 | 0.999 | 0.999 | 0.999 | 0.973 | 1 |

Table 1. Fingerprint similarity of 10 batches of Sini Decoction.
